# HYDIN mutation status as a potential predictor of immune checkpoint inhibitor efficacy in melanoma

**DOI:** 10.18632/aging.204925

**Published:** 2023-08-17

**Authors:** Liu Li, Kuang Tianrui, Li Chunlei, Qiu Zhendong, Chen Xiaoyan, Deng Wenhong

**Affiliations:** 1 Department of General Surgery, Renmin Hospital of Wuhan University, Wuhan, Hubei Province, China

**Keywords:** HYDIN, immune checkpoint inhibitors, melanoma, biomarker, prognosis

## Abstract

Background: The advent of immune checkpoint inhibitors (ICIs) has altered the outlook for cancer treatment. The estimation of predictive biomarkers could contribute to maximizing the benefits from ICIs treatment. Here, we explored the association between HYDIN mutations (HYDIN-MUT) in melanoma and ICIs efficacy.

Methods: Clinical data and sequencing data from published studies were utilized to assess the association between HYDIN-MUT and the efficacy of ICIs treatment in melanoma patients.

Results: Compared to other tumor types, HYDIN (36.14%) has the highest mutation rate in melanoma patients. In the anti-PD-1 treated cohort (n = 254), the HYDIN-MUT patients had a longer OS after ICIs treatment than the HYDIN wild-type (HYDIN-WT) patients (HR = 0.590 [95% CI, 0.410-0.847], P = 0.004); the objective response rate (ORR) and durable clinical benefit (DCB) were increased in patients with HYDIN-MUT (ORR = 46.25, DCB = 56.00%) compared to patients with HYDIN-WT (ORR = 30.99%, DCB = 42.76%) (ORR: P = 0.019; DCB: P = 0.060). In the anti-CTLA4 treated cohort (n = 174), HYDIN-MUT patients achieved significantly longer OS than HYDIN-WT patients (HR = 0.549 [95% CI, 0.366-0.823], P = 0.003); the proportion of ORR and DCB in HYDIN-MUT patients was significantly higher than that in HYDIN-WT patients (ORR 40.54% vs. 14.42%, P = 0.031; DCB 45.76% vs. 22.22%, P = 0.002). Further gene set enrichment analysis demonstrated that DNA repair and anti-tumor immunity were significantly enhanced in HYDIN-MUT patients.

Conclusions: HYDIN mutations are a potential predictive biomarker of ICIs efficacy in melanoma patients.

## INTRODUCTION

Melanoma is an aggressive malignancy caused by the uncontrolled proliferation of abnormal melanocytes. The progress of melanoma is rapid and its prognosis is very poor, with a 5-year survival rate of 20% [1, 2]. In recent years, although great progress has been made in traditional treatments such as radiotherapy, surgery, and chemotherapy, the efficacy is not satisfactory, with an overall response rate of less than 20%, owing to resistance and side effects [3]. In contrast, immunotherapy, especially immune checkpoint inhibitors (ICIs), can remarkably prolong overall survival (OS) and improve the prognosis of patients. Unfortunately, in clinical practice, only a minority of patients achieved durable responses with ICIs [4]. Biomarkers enable the identification of patients who are sensitive to ICIs, the prevention of unnecessary adverse effects in patients who are not sensitive to ICIs, and the reduction of patients’ financial burden. Considering these factors, it is necessary to assess reliable predictive biomarkers to guide the clinical treatment process.

Currently, predictive markers for ICIs therapy in melanoma include lactate dehydrogenase (LDH) [5], tumor-infiltrating lymphocytes [6], programmed cell death protein 1 (PD-1) expression [7–9], tumor mutation burden (TMB) [10], the host’s gastrointestinal microbiome [11, 12], and body-mass index [13], but these markers are not sufficient to predict prognosis. Recently, melanoma genomics-based gene mutation research has become a popular approach for biomarker discovery. For example, Johnson et al. demonstrated that melanoma patients with NRAS mutations had a higher response rate to first-line ICIs treatment with ipilimumab or anti-PD-L1 antibody than those with wild type [14]. It was also noted that melanoma patients with ROS1 mutations yielded longer OS from ICIs [15]. These results suggest that certain genetic mutations may be reliable predictive markers of ICIs efficacy in melanoma patients.

The human HYDIN gene encodes a protein that may be involved in cilia motility. It is known that mutations in this gene cause autosomal recessive primary ciliary dyskinesia-5, a disorder characterized by the accumulation of cerebrospinal fluid in the ventricles of the brain [16, 17]. Besides, HYDIN mutations have been found in breast cancer [18]. Yim et al. analyzed 45 cases of pure mucinous breast cancer and found that HYDIN (88%) possessed the most common somatic mutated rate [19]. Interestingly, back in 2013, Laske et al. [20] revealed a frequent and coordinated adaptive immune response to HYDIN mutations in tumor patients and first proposed HYDIN as a new cancer-associated antigen. However, studies on HYDIN in tumors are not well studied, and its relationship with ICIs therapy has not been studied. Thus, we intend to systematically investigate the association between HYDIN mutations and ICIs efficacy in melanoma patients.

## MATERIALS AND METHODS

### The ICIs treatment cohort

To assess the predictive value of HYDIN mutations, we conducted a comprehensive search for clinical studies of melanoma patients treated with ICIs using the PubMed Database. After reading the title, abstract, and full text of the search results, six published articles with mutational data and response data were selected [21–26]. However, the HYDIN gene was not included in the commercial targeted sequencing panel of Samstein et al. [23] and, therefore, five articles were ultimately included in our study. The whole-exome sequencing (WES) data and annotated clinical data from Snyder et al. [21], Allen et al. [22], Hugo et al. [26], and Liu et al. [24], were collected on the cBioPortal website (MSKCC, NEJM 2014; DFCI, Science 2015; UCLA, Cell 2016; DFCI, Nature Medicine 2019). The data of Riaz et al. [25] were obtained from the Supplementary Material of his article. The combined cohort of Riaz et al., Hugo et al., and Liu et al., using anti-PD-1 treatment, was defined as the anti-PD-1 treated cohort. The combined cohort of Snyder et al. and Allen et al., using anti-cytotoxic T-lymphocyte associated protein 4 (CTLA4) treatment, was considered the anti-CTLA4 treated cohort. Tumors with and without nonsynonymous somatic mutations of HYDIN were defined as HYDIN-mutant (HYDIN-MUT) and HYDIN-wildtype (HYDIN-WT), respectively.

### The cancer genome atlas (TCGA) cohort

The “TCGA PanCancer Atlas Studies” module in the cBioPortal database was utilized to estimate the frequency of mutations in HYDIN in pan-cancer. The “Skin Cutaneous Melanoma (TCGA, PanCancer Atlas)” dataset in the cBioPortal database was used to explore the details of HYDIN mutations in melanoma and the impact of HYDIN mutations on the prognosis of melanoma patients. The simple nucleotide variation, transcriptome profiling, and clinical data of the TCGA-SKCM cohort were acquired from GDC (https://portal.gdc.cancer.gov/). Next, a Perl script was used to collate the non-synonymous mutation information in the TCGA-SKCM cohort and to plot the gene mutation panorama using the GenVisR package.

### Measurement of clinical outcomes

Response Evaluation Criteria in Solid Tumors (RECIST) version 1.1 was used to estimate the objective response rate (ORR) [26]. Complete response, partial response, or stable disease (SD) lasting longer than 6 months was considered a durable clinical benefit (DCB); progression of disease or SD lasting less than 6 months was defined as no durable benefit (NDB) [27]. The TMB (nonsynonymous) and neoantigen load (NAL) data of Snyder et al., Allen et al., Hugo et al., and Liu et al. were obtained from cBioPortal. The TMB data of Riaz et al. were discarded as they differed from the definition of cBioPortal, and we only included the NAL data from Riaz et al.

### Pathway enrichment analysis

TCGA-SKCM cohort information was organized using Perl scripts. The gene set enrichment analysis (GSEA) was conducted based on HYDIN mutation status using the Molecular Signatures Database (MSigDB) of c2 (c2.cp.kegg.v7.2.symbols.gmt, c2.cp.reactome.v7.2.symbols.gmt) and c5 (c5.go.bp.v7.2.symbols.gmt) by GSEA 3.0.

### Statistical analysis

Categorical variables were estimated by the chi-square or Fisher’s exact test. For comparing continuous variables, the Mann-Whitney U test was utilized. The data were analyzed using SPSS 26.0. GraphPad Prism 8 is used for bar charting. Survival descriptions were displayed by Kaplan-Meier curves, and *P* values were determined by log-rank tests. Hazard’s ratio (HR) was calculated by a cox proportional risk and logistic regression model. In multivariate analysis, any covariates with a *P* value < 0.1 in univariate studies were considered. Stata 16.0 was used for the meta-analysis. The chi-squared test was used to determine statistical heterogeneity among the studies. *P* > 0.1 and I^2^ < 50% indicated low heterogeneity, and a fixed-effect model was employed. The tests of Egger and Begg were employed to evaluate publication bias. A sensitivity analysis was implemented to assess the stability of the results by excluding each study independently. *P* ≤ 0.05 was considered statistically significant.

### Availability of data and materials

All of the data we used in this study were publicly available as described in the “Methods” section.

## RESULTS

### Mutation profile of HYDIN in melanoma

**Figure 1:**
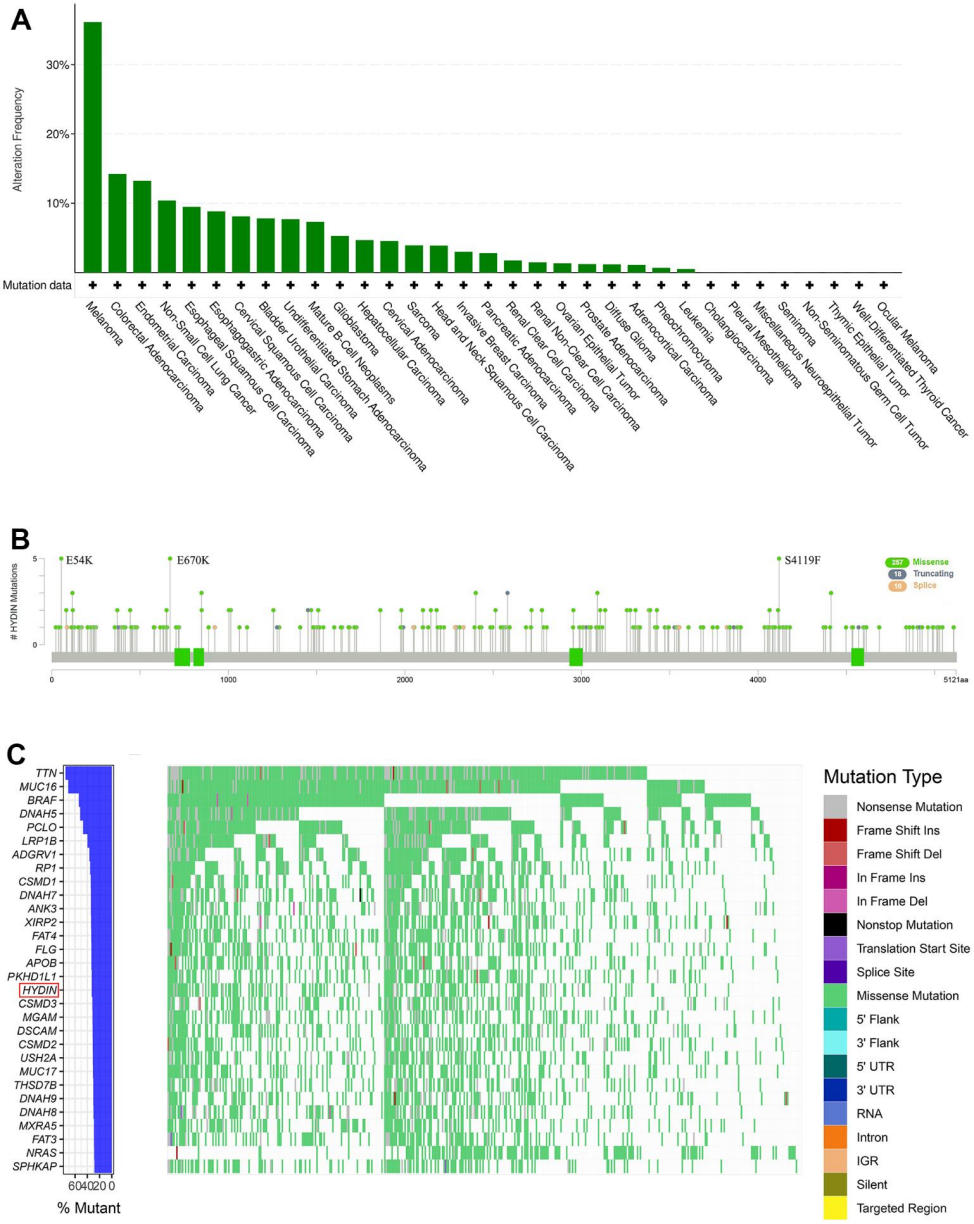
**Mutation profile of HYDIN in melanoma.** (**A**) Prevalence of HYDIN mutations across various cancers. (**B**) Mutation diagram of HYDIN in melanoma. (**C**) The gene mutation panoramas of the TCGA-SKCM cohort.

We estimated the mutation profile of HYDIN across various cancers using the cBioPortal database. The results revealed that 670 of the 10429 patients included in TCGA had mutations in HYDIN (6.42%), Notably, the highest incidence of HYDIN mutations was found in melanoma patients, with a rate of 36.14% (159/438) (Figure 1A). The types, sites, and case numbers of the HYDIN mutations in melanoma were further shown in Figure 1B. A total of 285 mutation sites (including 257 missenses, 18 truncating, and 10 splice) were found in HYDIN through the cBioPortal database. The most frequent protein changes were E54K, E670K, and S4119F. Besides, the gene mutation panorama was displayed in Figure 1C. We found the HYDIN mutations ranked 17th in the frequency of mutations in melanoma patients.

### HYDIN mutations were related to better clinical outcomes in the anti-PD-1 treated cohort

**Figure 2:**
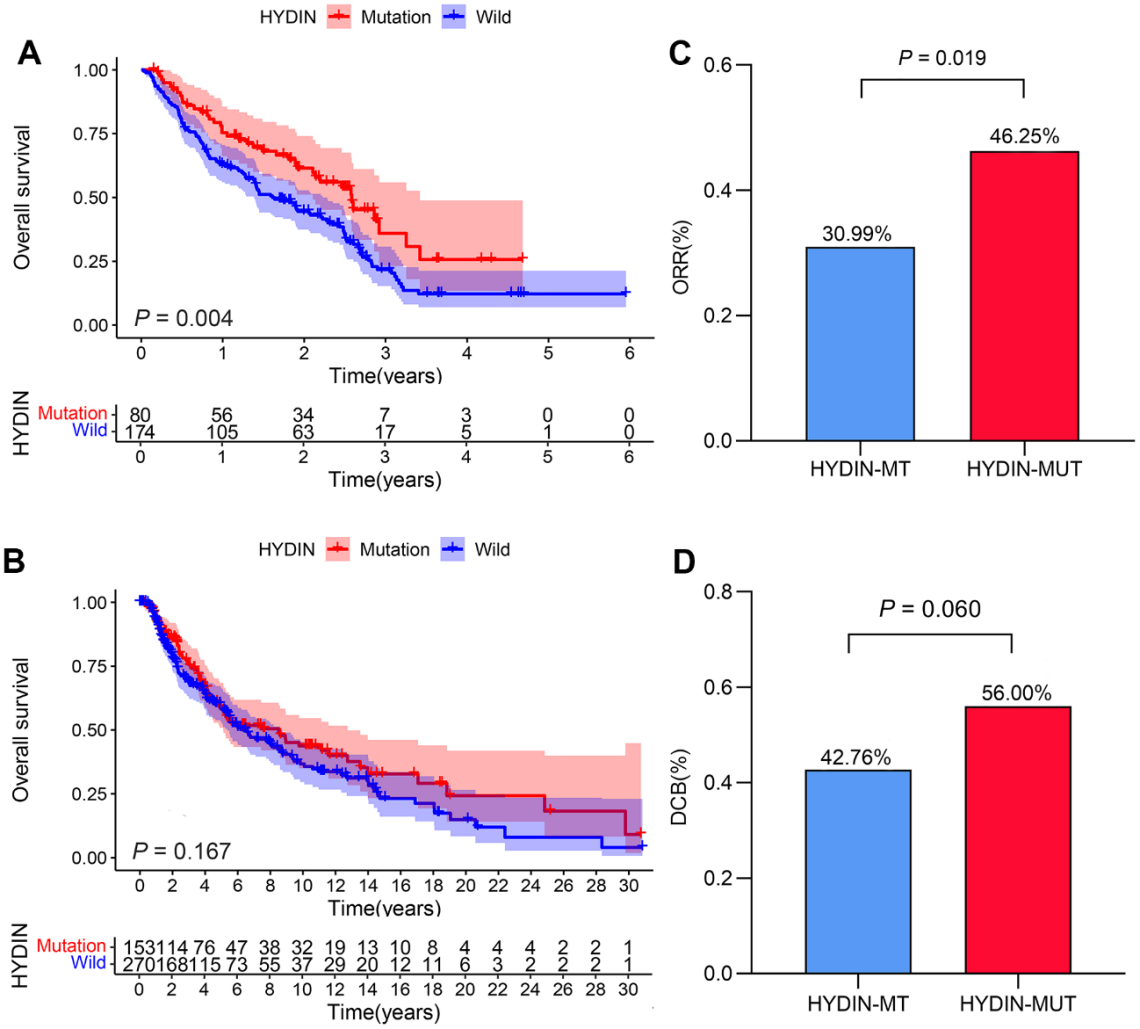
**HYDIN mutations were related to better clinical outcomes in the anti-PD-1 treated cohort.** The Kaplan–Meier curves of overall survival in the anti-PD-1 treated cohort (**A**) and the TCGA-SKCM cohort (**B**). Histogram depicting proportions of ORR (**C**) and DCB (**D**) in HYDIN-MUT and HYDIN-WT patients in the anti-PD-1 treated cohort. DCB, durable clinical benefit; ORR, objective response rate; HYDIN-MUT, HYDIN mutations; HYDIN-WT, HYDIN wild-type; Anti-PD-1, anti-programmed cell death protein 1.

Due to the high prevalence of HYDIN mutations, we investigated its predictive usefulness for the efficacy of ICIs treatment in the anti-PD-1 treated cohort. The baseline patient characteristics of the anti-PD-1 treated cohort are shown in Table 1. In an analysis of melanoma patients by gender, age, and M stage, it was found that male patients had a higher proportion of HYDIN mutations. Remarkably, the HYDIN-MUT patients had a longer OS after anti-PD-1 therapy than the HYDIN-WT patients (Figure 2A, median OS: 31.00 months vs. 19.67 months, HR = 0.590 [95% CI, 0.410–0.847], *P*_log rank_ = 0.004). Next, we explored the prognostic impact of HYDIN mutations in the non-anti-PD-1 treated patients using the TCGA data to clarify if the predictive effect of HYDIN mutations on anti-PD-1 was affected by itself. As shown in Figure 2B, there was no remarkable difference in OS between HYDIN-MUT patients and HYDIN-WT patients (HR = 0.819 [95% CI, 0.616–1.088], *P*_log rank_ = 0.167), which confirmed that HYDIN mutations can be used as potential predictive biomarkers for anti-PD-1 treatment of melanoma patients.

**Table 1:** Patient characteristics stratified by HYDIN status.

**Characteristic**	**Discovery cohort**				**Validation cohort**			
	**HYDIN-MUT**	**HYDIN-WT**	**Total**	***P*-value**	**HYDIN-MUT**	**HYDIN-WT**	**Total**	***P*-value**
**No. of patients**	80	174	254		60	114	174	
**Gender**								
Male	48	62	110	0.004	41	76	117	0.824
Female	16	55	71		19	38	47	
NA	16	57	73					
**Age group (years)**								
≤ 60	13	4	17	0.173	23	58	81	0.555
> 60	10	10	20		37	56	93	
NA	57	160	217		0	0	0	
**M stage**								
M0, M1a, M1b	16	43	59	0.550	20	32	52	0.471
M1c	55	121	176		40	82	122	
IIIC	3	7	10		0	0	0	
NA	6	3	9		0	0	0	
**Treatment**								
Anti-PD-1	80	174	254	-	0	0	0	-
Anti-CTLA4	0	0	0		60	114	174	
**Treatment response**								
Response	37	53	90	0.019	16	15	31	0.031
Nonresponse	43	118	161		40	89	129	
NA	0	3	3		4	10	14	
**Durable clinical benefit**								
DCB	42	65	107	0.060	27	24	51	0.002
NDB	33	87	120		32	84	116	
NA	5	22	27		1	6	7	

HYDIN-MUT, HYDIN mutation; HYDIN-WT, HYDIN wild type; DCB, durable clinical benefit; NDB, no durable benefit; PD-1, programmed cell death protein 1; CTLA4, cytotoxic T-lymphocyte associated protein 4.

The available response data were further evaluated in the cohort of anti-PD-1 therapy. Interestingly, we found that the ORR and DCB of patients were increased in patients with HYDIN-MUT (ORR = 46.25%; DCB = 56.00%) compared to patients with HYDIN-WT (ORR = 30.99%; DCB = 42.76%) (ORR: *P* = 0.019; DCB: *P* = 0.060; Figure 2C, 2D).

### HYDIN mutations were associated with a better prognosis in the anti-CTLA4 treated cohort

**Figure 3:**
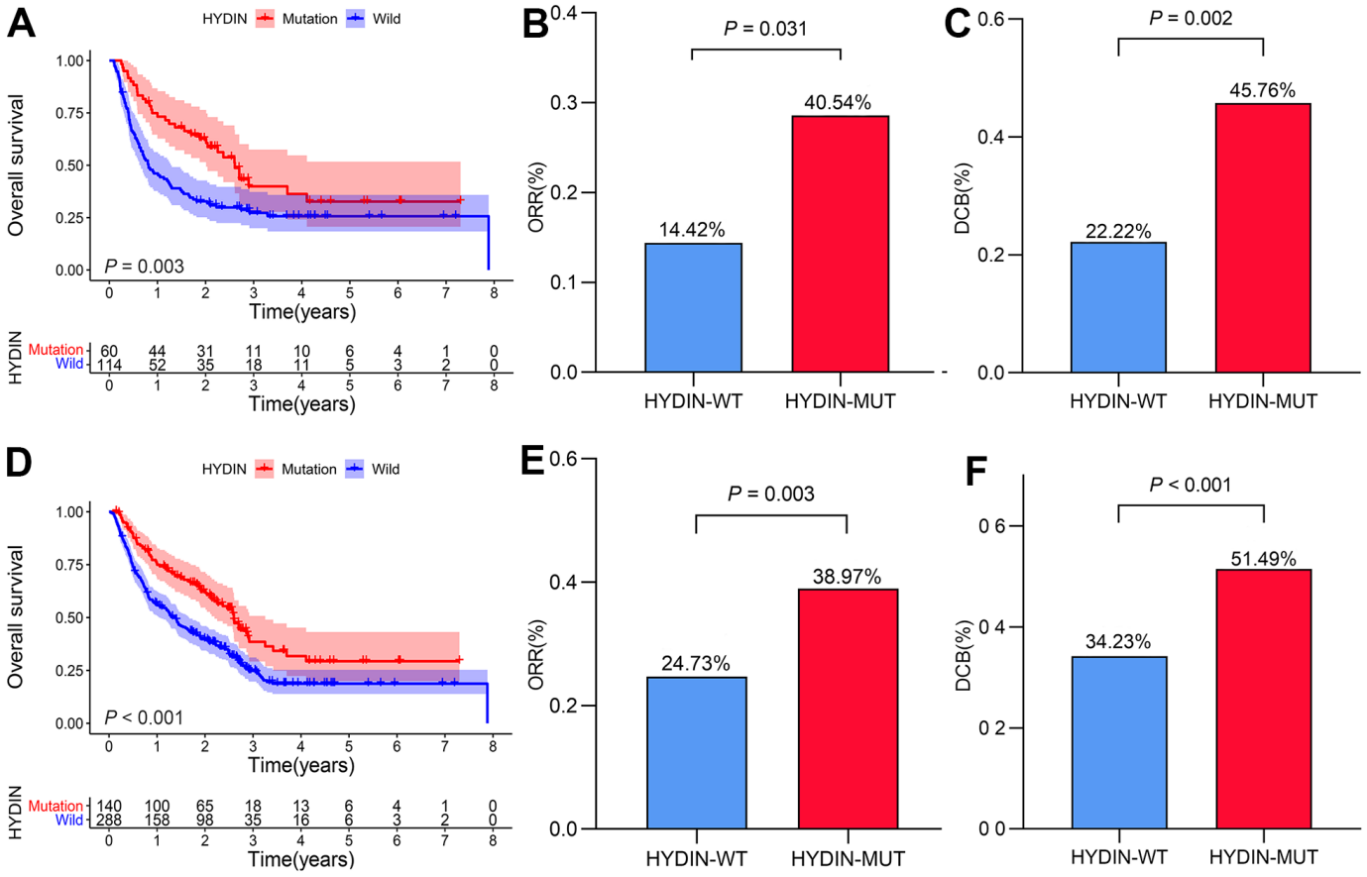
**Validation of the prognostic value of HYDIN status.** The Kaplan–Meier curve of overall survival in the anti-CTLA4 treated cohort (**A**). Histogram depicting proportions of ORR (**B**) and DCB (**C**) in HYDIN-MUT and HYDIN-WT patients in the anti-CTLA4 treated cohort. Kaplan–Meier curves of overall survival in the total ICIs treatment cohort (**D**). Histogram depicting proportions of ORR (**E**) and DCB (**F**) in HYDIN-MUT and HYDIN-WT patients in the total ICIs treatment cohort. DCB, durable clinical benefit; ORR, objective response rate; HYDIN-MUT, HYDIN mutations; HYDIN-WT, HYDIN wild-type; anti-CTLA4, anti-cytotoxic T-lymphocyte associated protein 4.

We further conducted a survival analysis in the anti-CTLA4 treated cohort. Baseline patients’ characteristics are provided in Table 1. The results showed that HYDIN mutations did not differ significantly by gender, age, and M stage. The survival analysis was consistent with the anti-PD-1 treated cohort; that is, patients with HYDIN-MUT had a longer OS than patients with HYDIN-WT (Figure 3A, median OS: 31.30 months vs. 9.77 months, HR = 0.549 [95% CI, 0.366-0.823], *P*_log rank_ = 0.003). Similarly, there was a higher proportion of ORR and DCB in HYDIN-MUT patients than in HYDIN-WT patients (Figure 3B, 3C, ORR: 40.54% vs. 14.42%, *P* = 0.031; DCB: 45.76% vs. 22.22%, *P* = 0.002).

### Validation of the prognostic value of HYDIN status in the entirely ICIs-treated cohort

We combined the survival and response data from the above two cohorts of 428 patients, and the results continued to show that patients with HYDIN mutations had longer OS (Figure 3D, median OS: 31.20 months vs. 16.57 months, HR = 0.575 [95% CI, 0.439-0.753], *P*_log rank_ < 0.001) and higher ORR and DCB rates (Figure 3E, 3F, ORR: 38.97% vs. 24.73%, *P* = 0.003; DCB: 51.49% vs. 34.23%, *P* < 0.001).

**Figure 4:**
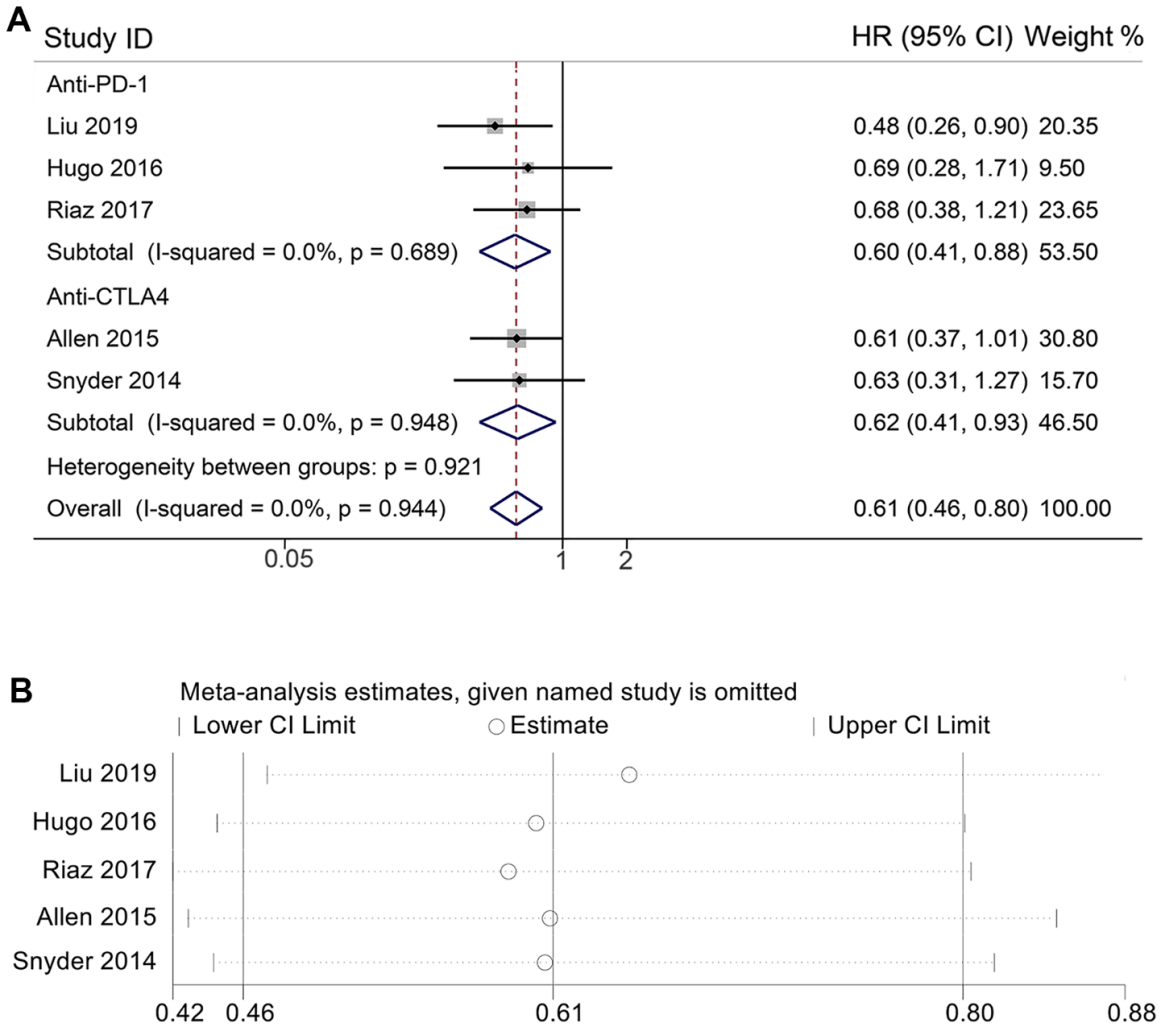
**Meta-analysis of HYDIN mutations and overall survival.** (**A**) Forest plot of the relationship between HYDIN mutations and overall survival in melanoma. (**B**) Sensitivity analysis of overall survival. HR, Hazard ratio; CL, Confidence interval; anti-CTLA4, anti-cytotoxic T-lymphocyte associated protein 4; Anti-PD-1, anti-programmed cell death protein 1.

Moreover, a meta-analysis was conducted to explore heterogeneity among the five included studies (Figure 4A). The test for heterogeneity showed no heterogeneity in the anti-PD-1 treated cohort (*P* = 0.689, I^2^ = 0.0%), the anti-CTLA4 treated cohort (*P* = 0.948, I^2^ = 0.0%), and the total ICIs treatment cohort (*P* = 0.944, I^2^ = 0.0%). The meta-analysis results also reaffirmed that HYDIN-MUT predicted a better prognosis for ICIs treatment (anti-PD-1 cohort, HR = 0.598 [95% CI, 0.408-0.877], *P* = 0.008; anti-CTLA4 cohort, HR = 0.615 [95% CI, 0.408-0.928], *P* = 0.020; total ICIs treatment cohort, HR = 0.606 [95% CI, 0.458-0.802], *P* < 0.001). Furthermore, Begg’s test and Egger’s test show no publication bias (Egger’s test: P = 0.780, Begg’s test: P = 0.806). To assess the impact of each study on the overall meta-analysis, we performed a sensitivity analysis using the leave-one-out method. The results revealed that no single study was able to significantly impact the pooled HR of OS (Figure 4B). Thus, we believe that our conclusion may be stable, suggesting that HYDIN mutations may be a useful predictor and HYDIN-MUT patients may benefit more from ICIs.

### HYDIN-MUT was an independent predictor of ICIs therapy prognosis

Univariate and multivariate Cox regression analyses were utilized to estimate the independent predictive value of HYDIN status in the total ICIs treatment cohort. Univariate analysis indicated that HYDIN status, M stage, TMB, and NAL were associated with OS (*P* < 0.05), while gender and age were not significantly related to OS (Table 2). After adjusting for M stage, TMB, and NAL by multivariate analysis, we found that HYDIN-MUT was an independent predictive biomarker of better prognosis (Table 2, HR = 0.658 [95% CI, 0.444-0.977], *P* = 0.038), while the M stage was independently related to poor survival (Table 2, HR =1.774 [95% CI, 1.189-2.646], *P* = 0.005). Furthermore, multivariate logistic regression analyses also confirmed that HYDIN was independently associated with higher ORR (Table 2, HR = 2.289 [95% CI, 1.108-4.762], *P* = 0.025) and DCB (Table 2, HR = 2.336 [95% CI, 1.203-4.537], *P* = 0.012).

**Table 2:** Univariate and multivariate analyses to predict clinical outcomes.

**Factor**	**Univariate**		**Multivariate**	
	**Hazard ratio (95% CI)**	***P* value**	**Hazard ratio (95% CI)**	***P* value**
Overall survival				
HYDIN	0.545(0.379-0.785)	0.001	0.658(0.444-0.977)	**0.038**
TMB	0.531(0.377-0.748)	<0.001	0.680(0.421-1.098)	0.115
NAL	0.603(0.421-0.864)	0.006	0.572(0.521-1.434)	0.572
M Stage	1.732(1.168-2.569)	0.006	1.774(1.189-2.646)	**0.005**
Age	0.984(0.700-1.385)	0.928		
Gender	0.838(0.578-1.195)	0.328		
Objective response rate				
HYDIN	2.828(1.469-5.444)	0.002	2.289(1.108-4.762)	**0.025**
TMB	2.509(1,278-4.928)	0.008	1.453(0.544-3.876)	0.456
NAL	2.337(1.222-4.472)	0.010	1.255(0.496-3.173)	0.631
M Stage	0.729(0.366-1.450)	0.368		
Age	1.779(0.925-3.421)	0.084	1.284(0.622-2.651)	0.498
Gender	2.201(1.019-4.756)	0.045	2.095(0.934-4.699)	0.073
Durable clinical benefit				
HYDIN	3.067(1.687-5.575)	<0.001	2.336(1.203-4.537)	**0.012**
TMB	2.986(1.616-5.517)	<0.001	2.159(0.929-5.018)	0.074
NAL	2.329(1.291-4.201)	0.005	1.083(0.460-2.551)	0.855
M Stage	0.538(0.289-1.000)	0.050	0.516(0.266-1.003)	0.051
Age	0.765(0.428-1.368)	0.367		
Gender	0.850(0.455-1.587)	0.609		

Age (years): > 60 vs ≤ 60 (reference values); Gender: male vs female (reference values); M Stage: M1c vs M1b, M1a, M0 (reference values); TMB/NAL, top 50% vs 50% of the latter (reference values); CL, Confidence interval.

### Patients with HYDIN-MUT have an elevated TMB and NAL

**Figure 5:**
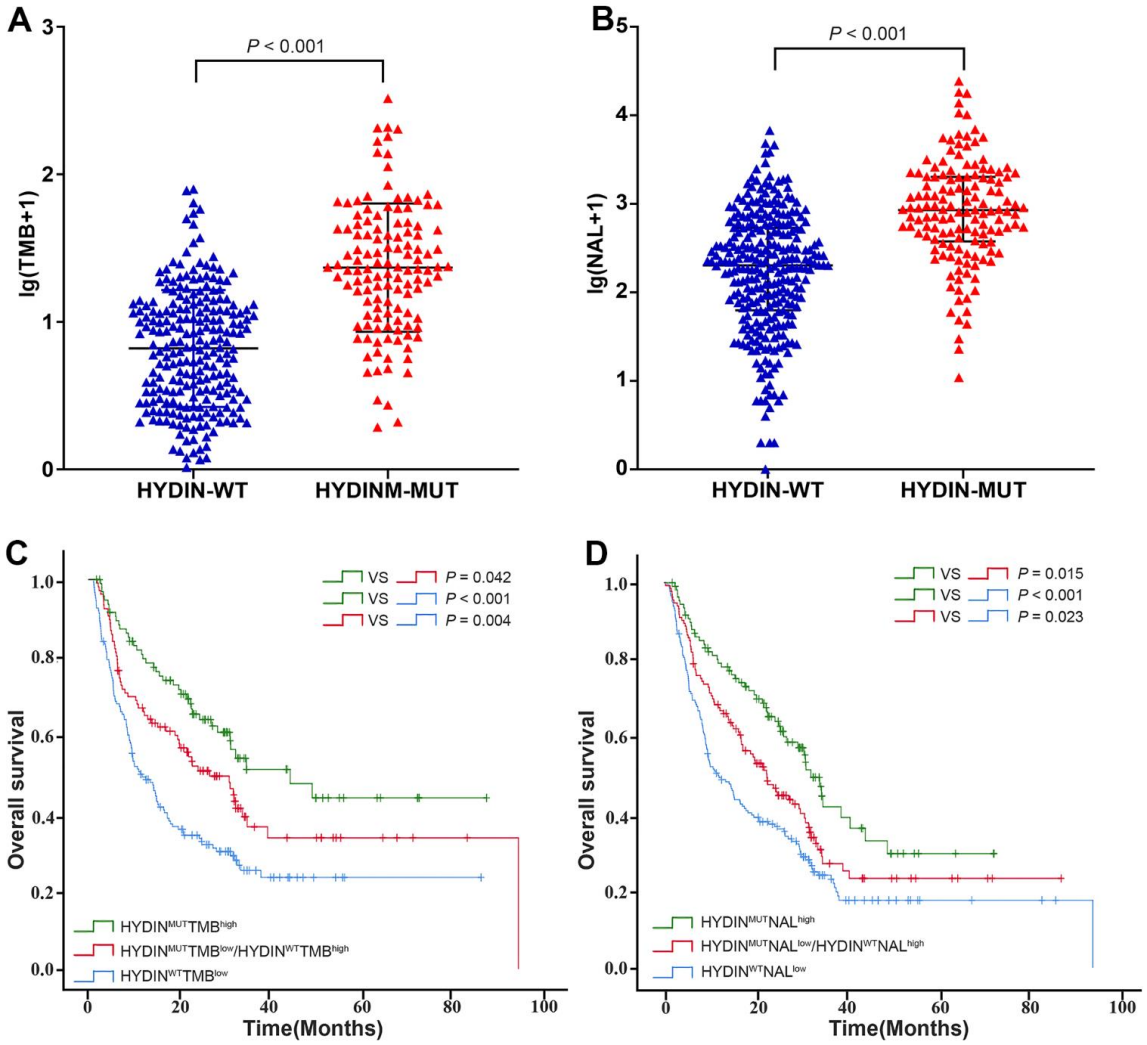
**Correlation between TMB, NAL, and HYDIN mutation status.** Distribution of TMB (**A**) and NAL (**B**) in the total ICIs treatment cohort. (**C**) The Kaplan-Meier curves comparing OS among HYDIN^MUT^TMB^high^, HYDIN^MUT^TMB^low^/HYDIN^WT^TMB^high^, and HYDIN^WT^TMB^low^ in the total ICIs treatment cohort. (**D**) The Kaplan-Meier curves comparing OS among HYDIN^MUT^NAL^high^, HYDIN^MUT^NAL^low^/HYDIN^WT^NAL^high^, and HYDIN^WT^NAL^low^ in the total ICIs treatment cohort. HYDIN-MUT, HYDIN mutations; HYDIN-WT, HYDIN wild-type.

To further explore why patients with HYDIN mutations are more likely to benefit from ICIs treatment, we next explored tumor immunogenicity. We found that in the total ICIs treated cohort, melanoma patients with the HYDIN-MUT had significantly higher TMB and NAL (Figure 5A, 5B), suggesting that the HYDIN mutations were associated with higher tumor immunogenicity. Based on HYDIN status and median TMB level, we separated them into three groups: HYDIN^MUT^ TMB^high^, HYDIN^MUT^TMB^low^/HYDIN^WT^TMB^high^, and HYDIN^WT^TMB^low^. As expected, the patients with HYDIN^MUT^TMB^high^ achieved the longest OS among all groups (Figure 5C, HYDIN^MUT^TMB^high^ vs. HYDIN^MUT^TMB^low^/HYDIN^WT^TMB^high^, median OS: 44.40 months vs. 26.7 months, HR = 0.661 [95% CI, 0.442-0.988], *P*_log rank_ = 0.042; HYDIN^MUT^TMB^high^ vs. HYDIN^WT^TMB^low^, median OS: 44.40 months vs. 11.433 months, HR = 0.412 [95% CI, 0.286-0.594], *P*_log rank_ < 0.001). The OS was also significantly longer in patients with HYDIN^MUT^TMB^low^/HYDIN^WT^TMB^high^ than in patients with HYDIN^WT^TMB^low^ (median OS: 26.7 months vs. 11.43 months, HR = 0.622 [95% CI, 0.453-0.865], *P*_log rank_ = 0.004).

The same analysis was conducted for NAL (Figure 5D). HYDIN^MUT^NAL^high^ patients had the longest OS among all groups (HYDIN^MUT^NAL^high^ vs. HYDIN^MUT^NAL^low^/HYDIN^WT^NAL^high^, median OS: 32.40 months vs. 22.71 months, HR = 0.653 [95% CI, 0.462-0.922], *P*_log rank_ = 0.015; HYDIN^MUT^NAL^high^ vs. HYDIN^WT^NAL^low^, median OS: 32.40 months vs. 11.87 months, HR = 0.412 [95% CI, 0.286-0.594], *P*_log rank_ < 0.001). HYDIN^MUT^NAL^low^/HYDIN^WT^NAL^high^ patients also have a much longer OS than HYDIN^WT^NAL^low^ (median OS: 22.71 months vs. 11.87 months, HR = 0.622 [95% CI, 0.453-0.856], *P*_log rank_ = 0.023). Thus, the higher TMB and NAL in HYDIN-MUT patients may be partly associated with their better response to ICIs therapy.

### GSEA analysis between HYDIN-MUT and HYDIN-WT

**Figure 6:**
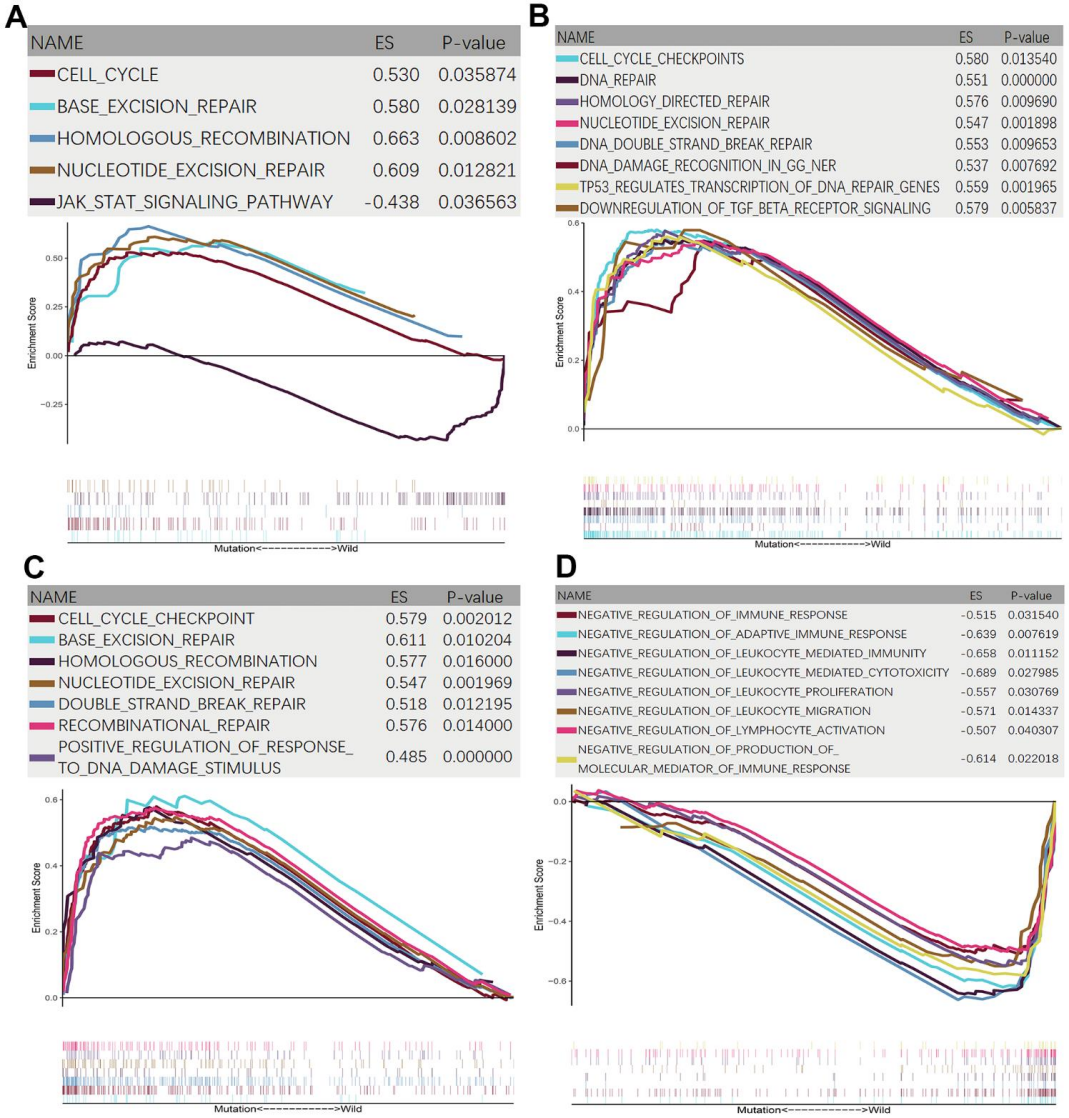
**GSEA enrichment analysis between HYDIN-MUT and HYDIN-WT.** (**A**) KEGG enrichment analysis. (**B**) Reactome enrichment analysis. (**C**, **D**) GO-BP enrichment analysis.

The results of enrichment analysis showed that several pathways varied significantly between HYDIN-MUT and HYDIN-WT patients. As shown in Figure 6, the enrichment analyses of KEGG, Reactome, and GO-BP results showed that cell cycle checkpoints, DNA repair (such as base excision repair, homologous recombination, nucleotide excision repair, double-strand break repair, and recombinational repair), and DNA damage recognition pathways were significantly upregulated in HYDIN-MUT patients (Figure 6A–6C). We also observed that TGF-β receptor signaling was significantly downregulated in HYDIN-MUT patients (Figure 6B). In contrast, immune response pathways (adaptive immune response, leukocyte mediated immunity, leukocyte mediated cytotoxicity, leukocyte proliferation, leukocyte migration, lymphocyte activation, production of molecular mediator of immune response) were significantly downregulated in HYDIN-WT patients (Figure 6D), and the Jak-STAT signaling pathway was significantly upregulated in HYDIN-WT patients (Figure 6A).

## DISCUSSION

Malignant melanoma is one of the most aggressive and highly drug-resistant malignancies of melanocytes and can occur throughout the body. It originates from melanocytes in the skin, mucosa and uvea [28]. The incidence of melanoma has been reported to be on the rise in recent years [29]. However, since the introduction of novel therapeutic agents, such as immunotherapy with immune checkpoint inhibitors (ICIs), survival rates for patients with advanced melanoma have radically improved [30–32]. Immune checkpoints, proteins expressed by T cells, regulate T cell function and act as gatekeepers of the immune response to prevent autoimmunity. Immune checkpoint inhibitors are designed to release damaged T cells and thus induce an anti-tumor response [33, 34].

The HYDIN gene is a gene involved in encoding a protein related to ciliary motility. Mutations in this gene lead to autosomal recessive primary ciliary dyskinesia-5, which causes a disease characterized by intracerebroventricular cerebrospinal fluid accumulation [16, 17]. The abnormal motility of the mutant cilia greatly reduces or eliminates blood flow generated by the ventricular meningeal cilia, and this lack of flow may be the underlying cause of hydrocephalus in these mutants [35]. Interestingly, mutations in the HYDIN gene have recently been identified in tumors as well. Yim et al. [18] analyzed 45 cases of pure mucinous breast cancer and found the highest rate of somatic mutations in HYDIN. In addition, Laske et al. [20] revealed frequent and coordinated adaptive immune responses to HYDIN mutations in tumor patients and proposed for the first time that HYDIN is a novel cancer-associated antigen. However, HYDIN has not been adequately studied in tumors, and its relationship with the treatment of ICIs has not been investigated.

In this work, we explored the correlation between HYDIN mutations and response in melanoma patients with ICIs treatment. We found that the HYDIN mutations were significantly related to better prognosis in ICIs-treated patients. Notably, univariate and multivariate analyses further confirmed that the HYDIN mutations were an independent positive predictor. Our study will enable a re-examination of the value of this gene as a new predictive molecule for melanoma patients. More importantly, the high mutation rate of HYDIN in melanoma patients (36.14%) means that more beneficiaries can be identified for precision treatment.

TMB can effectively assess overall mutational load and neoantigenic load [36, 37]. In 2014, Snyder et al. reported for the first time a positive association between the response rate to ICIs therapy and TMB in melanoma patients [2]. Currently, some studies have found that TMB can effectively predict the response to ICIs in various cancer types, including melanoma [27, 38–42]. It is well known that ICIs therapy aims to inhibit cancer progression by activating the immune system to kill cancer cells. By choosing neoantigens generated by tumor-specific mutations, an effective tumor-specific immune response can be induced and immune tolerance can be reduced [43–46]. Non-synonymous cellular mutations can generate neoantigens, which are key elements required for successful ICIs therapy [47–49]. Current clinical studies have also demonstrated that NAL is associated with benefits in melanoma patients with ICIs treatment [22]. In the present study, we revealed that TMB and NAL levels were much higher in HYDIN-MUT patients than in HYDIN-WT patients. To further explore whether the benefit of ICIs treatment in HYDIN-MUT patients was partly due to these patients having higher levels of TMB and NAL, we performed a multivariate Cox analysis and a multi-group survival analysis. Interestingly, we found that HYDIN-MUT was an independent predictor of patient benefit, and patients with HYDIN^MUT^TMB^high^ or HYDIN^MUT^NAL^high^ had a much better prognosis than those with HYDIN^MUT^TMB^low^/HYDIN^WT^TMB^high^ or HYDIN^MUT^NAL^low^/HYDIN^WT^NAL^high^.

Notably, GSEA results indicated that cell cycle checkpoints, DNA repair pathways, and DNA damage recognition pathways were significantly enriched in HYDIN-MUT patients. This means that in HYDIN-MUT patients, an increase in TMB and NAL was followed by an upregulation in DNA self-repair. As we know, DNA is under constant attack and a typical human cell undergoes an estimated 70,000 damages per day [50]. Defects in DNA damage repair may cause an accumulation of mutations and an increase in cell death, promoting cancer or aging, respectively. However, intricate repair pathways have evolved to address the persistent issue of DNA damage, which in turn inhibits the emergence of tumors [51]. Furthermore, our multiple GSEA enrichment results confirmed the presence of high levels of anti-tumor immunity in HYDIN-MUT patients, contributing to the efficacy of ICIs therapy. As for the Jak-STAT pathway, the fundamental role of its perturbation in tumorigenesis, particularly in melanoma, has been established over the last decade. STAT3 was constitutively activated in 40% of primary cutaneous melanomas and in 60% of melanomas that metastasized to regional lymph nodes [52, 53]. STAT3 activation can prevent apoptosis and support the proliferation of melanoma cells [52, 53]. Overexpression of TGF-β and loss of growth inhibition have now been described in melanoma [54]. Several studies have indicated that increased levels of TGF-β expression correlated with tumor progression [54, 55], and that downregulation of the TGF-β receptor can inhibit the pro-carcinogenic effects of the overexpression of TGF-β. Therefore, the better prognosis of HYDIN-MUT patients may be partly attributed to the downregulation of the TGF-β receptor and Jak-STAT pathways.

There are also some limitations to this study. First, the predictive significance of HYDIN mutations and the specific molecular mechanisms behind them have not been fully validated experimentally, so a more comprehensive biological mechanism remains to be elucidated in future studies. Secondly, due to the retrospective nature of the post-hoc analysis, there is still a need to validate HYDIN mutations in prospective clinical trials. As for limitations, the retrospective pooled estimation methodology may introduce some biases. The limitation of retrospective studies could be greatly minimized by the large sample size (5 independent studies involving 428 patients) in this study, by which the experimental characteristics might be balanced, such as race, the sequencing platform, etc. Besides, our meta-analysis also confirmed that there was no heterogeneity between studies, which provides strong support for our pooled estimates. Importantly, these limitations do not preclude the favorable clinical outcomes derived from ICIs treatment in HYDIN-MUT patients. Unlike continuous variables such as TMB whose optimum cutoff value remains controversial, HYDIN mutations are easily detected by High-Throughput Sequencing and classify patients into two groups that are related to ICIs therapy response.

## CONCLUSIONS

HYDIN mutations successfully predict better clinical outcomes in ICIs-treated melanoma patients, indicating that HYDIN mutations could be a potential predictive biomarker for ICIs in melanoma patients.
